# TASP1 Promotes Proliferation and Migration in Gastric Cancer via EMT and AKT/P-AKT Pathway

**DOI:** 10.1155/2021/5521325

**Published:** 2021-04-29

**Authors:** Xue-Mei Wan, Xue-Lei Zhou, Yong-Jun Du, Hui Shen, Zhengxia Yang, Yanhong Ding

**Affiliations:** ^1^Department of Infectious Diseases, Hospital of Chengdu University of Traditional Chinese Medicine, No. 39 Shi-er-qiao Road, Chengdu 610072, Sichuan Province, China; ^2^Department of Gastroenterology, Hospital of Chengdu University of Traditional Chinese Medicine, No. 39 Shi-er-qiao Road, Chengdu 610072, Sichuan Province, China; ^3^Department of Proctology, Hospital of Chengdu University of Traditional Chinese Medicine, No. 39 Shi-er-qiao Road, Chengdu 610072, Sichuan Province, China; ^4^The Affiliated Huai'an Hospital of Xuzhou Medical University and The Second People's Hospital of Huai'an, No. 62, Huaihai Road (S.), Huaian 223002, China

## Abstract

Threonine aspartase 1 (TASP1) was reported to function in the development of cancer. However, the regulatory mechanism of TASP1 in gastric cancer (GC) remains unclear. In this study, we determined the expression of TASP1 in tissues of GC patients, GC cells by qRT-PCR, and western blot and assessed the relationship between TASP1 and GC cell proliferation and migration via CCK-8 and transwell assay. It was found that the expression of TASP1 in GC tissues or GC cell lines was significantly higher than that in normal adjacent tissues or normal cells. The proliferation and migration of GC cells were inhibited upon TASP1 knockdown. Mechanism investigation revealed that TASP1 promoted GC cell proliferation and migration through upregulating the p-AKT/AKT expression. TASP1 induced GC cell migration via the epithelial -mesenchymal transition (EMT) pathway. In conclusion, TASP1 promotes GC progression through the EMT and AKT/p-AKT pathway, and it may serve as a new potential biomarker and therapeutic target for GC.

## 1. Introduction

Gastric cancer (GC) ranks third in the tumor mortality rate over the world [1]. Most GC patients have no obvious symptoms in the early stage and then are diagnosed at an advanced stage due to the deficiency of clinically effective markers [2]. At present, common tumor markers used for the diagnosis of GC include carcinoembryonic antigen, carbohydrate antigen 19-9, carbohydrate antigen 72-4, tumor necrosis factor-*α*, interleukin-6, and interleukin-8. However, the sensitivity and specificity of these biomarkers are still low [3]. Recent studies reported several novel biomarkers such as pepsinogen, *α*-fetoprotein, M2-pyruvate kinase, vascular adhesion protein-1, microRNA, long noncoding RNA, and circular RNA, but their effectiveness and accuracy remain under investigation [2, 4]. In the treatment of gastric cancer, apart from minimally invasive gastrectomy and radiotherapy, chemotherapy drugs are approved for the clinical treatment of GC patients, whose survival rate is low yet [5–10]. Therefore, there is still an urgent need to discover new biomarkers and therapeutic targets.

Proteolysis is a regulatory pathway in biological processes such as apoptosis [11]. The targeted proteases in proteolysis are also reported to play multiple roles in carcinogenesis [12]. Threonine aspartase 1 (TASP1), a particularly conserved protease that cleaves mixed lineage leukemia 1 (MLL), regulates the expression of homeotic genes [13] and is involved in the occurrence of cancer [14, 15]. Previous studies found that TASP1 was highly expressed in cancer cells [12, 16], and TASP1 downregulation could promote the apoptosis and inhibit the proliferation of cancer cells [13, 17]. TASPIN is an aspartase 1 inhibitor that can inhibit the development of breast cancer and brain cancer cells, thus exerting therapeutic effect [16, 18]. However, the mechanism of TASP1 action on GC is not clear.

The threonine-protein kinase (AKT) signaling pathway is crucial for a cellular activity, such as cell proliferation, survival, apoptosis, migration, and differentiation [19, 20]. The AKT signaling pathway has been observed to upregulate in GC [21]. It has been reported that p-AKT may be a prognosis indicator for GC patients [20].

Here, we measured the mRNA and protein levels of TASP1 in GC tissues and GC cells and evaluated the role of TASP1 in the proliferation and migration ability of GC cells. And we explored the possible mechanism of TASP1 mediating GC progression. The findings might provide new clues for determining the carcinogenic activity of TASP1 in GC.

## 2. Materials and Methods

### 2.1. The Tissue Collection

20 tissue samples of GC patients and 20 paired matched adjacent noncancerous tissues were obtained at the affiliated Huai'an hospital of Xuzhou Medical University (Jiangsu, China). The study was agreed to implement by the Huai'an Second People's Hospital Human Research Ethics Committee, and all volunteers in this study signed informed consent. Surgical resection underwent with all volunteers without any treatment. Fresh stomach tissue samples were quickly stored in liquid nitrogen and frozen.

### 2.2. Cell Culture

The normal human gastric mucosal cell GES1 was purchased from the ACTT Cell Bank. The human GC cell lines (BGC-803, AGS, SGC-7901, BGC-823) were purchased from the Shanghai Institutes for Biological Sciences (Shanghai, China). GES1 cells, and BGC-803 cells were cultured in DMEM medium with 10% fetal bovine serum and 1% penicillin-streptomycin (Gibco). AGS cells, BGC-823 cells, and SGC-7901 cells were cultured in RPMI-1640 medium with 10% fetal bovine serum and 1% penicillin-streptomycin (Gibco). Cells were put into a humidified incubator (37°C, 5% carbon dioxide).

### 2.3. Total RNA Isolation and Purification, Plasmid Construction, and Quantitative Real-Time PCR (qRT-PCR)

Using TRIzol Reagent (Invitrogen, Waltham, MA, USA) to isolate total RNA from tissues and cultured cells, then incubating the total RNA and 3 U/*μ*g exonuclease at 37°C for 40 minutes, total RNA was purified by RNeasy MinElute cleanup Kit (Qiagen), inserting the gene sequences containing short hairpin RNA (shRNA, 5′-TTCTCCGAACGTGTCACGT-3′, negative control) and TASP1 (5′-CAGAUUUUAUGCAACUAAA-3′) into pGMLV-SC5 lentiviral vectors (San Diego, CA, USA). The cells were infected with lentivirus for 48 hours when the ratio of MOI was 4, and then selected for 3 days with promycin (1 *μ*g/mL). The TASP1 (NM_017714) full-length cDNA sequence was amplified and cloned into the pCMVPuro02 expression vector and transfecting the plasmid into GC cells (SGC-7901, BGC-823) by ViaFect™ transfection reagent according to the manufacturer's instructions (Promega), then comparing the expression levels of transfected cells with empty vector transfected cells (MOCK).

Synthesizing cDNA from RNA was purified from tissues and cells with Reverse Transcription Kit (TaKaRa, Dalian, China). Then, the target gene was amplified using the SYBR-Green method (TaKaRa, Dalian, China). The product was detected with the StepOnePlus™ Real-Time PCR system (application biological system). GAPDH was used as the internal reference of TASP1 mRNA, and all operations were done by the manufacturer instructions.

### 2.4. Western Blot

The proteins were seperated in the cell lysate by SDS-PAGE and transferred to 0.45 *μ*m PVDF membrane (Millipore). Then, PVDF membrane was blocked with 5% skim milk and incubated with primary antibodies including anti-TASP1 (Abcam, Cambridge, MA, USA, ab63160), anti-E-cadherin (Cell Signaling Technology, #3195), anti-N-cadherin (Cell Signaling Technology, #13116), anti-vimentin (Cell Signaling Technology, #3295), anti-AKT (Cell Signaling Technology, #9272), anti-p-AKT (Cell Signaling Technology, Ser473, #4060), and anti-GAPDH (Cell Signaling Technology, Danvers, MA, USA, #2118). Next, proper secondary antibodies were added at room temperature, and the proteins were measured by enhanced chemiluminescence reagent and visualized by the Electrophoresis Gel Imaging Analysis System (DNR Bio-Imaging Systems, Jerusalem, Israel).

### 2.5. CCK-8

To detect the proliferation of cells, we use Cell Counting Kit-8 (CCK-8) (Dojindo Chemical Laboratory, Kumamoto, Japan). In 96-well plates, 100 *μ*L medium with transfected cells was added into each well, adding extra 10 *μ*L CCK reagent (Dojindo Chemical Laboratory, Kumamoto, Japan) at different time points (day 1, day 2, day 3, day 4, day 5) and culturing the cells at 37°C for 2 hours, then applying a microplate reader (Bio-TEK, Saxony, USA) for measuring the absorbance value to get a growth curve of GC cells at a wavelength of 450 nm.

### 2.6. Cell Colony Formation

In addition, to observe the colony formation of the GC cells, we inoculate SGC-7901 cells and BGC-823 cells in 6-well plates (1 × 10^4^ cells/well) supplemented with RPMI-1640 medium. After 10 days of cultivation, 0.1% crystal violet (Sigma-Aldrich) with 10% methanol was used to fix and stain the colonies (containing ≥50 cells), rinsing for three times with PBS before taking photos and counting the stained colonies.

### 2.7. Transwell Assay

300 *μ*L of cell suspension was added into the upper chamber of a crosswell plate (8 *μ*m size, Corning, New York, United States), and 700 *μ*L RPMI-1640 medium was added into the lower chamber. After incubated for 24 hours at 37°C, the cells were fixed with methanol for 10 min and stained with 0.1% crystal violet (Sigma-Aldrich) after removing the nonmigrated cells. Then, stained cells were counted in 5 randomly selected fields under microscope (Leica, Wetzlar, Germany).

### 2.8. Statistical Analysis

All data was analyzed with GraphPad Prism 7.04 software (Graphpad Software, Inc., San Diego, CA) in this study. The *t*-test was used to distinguish the statistical difference between the paired data of two groups. All data was tested at least 3 times independently. The results were shown as a way of mean ± standard deviation, and *p* < 0.05 suggested significant differences between two groups.

## 3. Results

### 3.1. TASP1 Is upregulated in GC Tissues and GC Cells

**Figure 1:**
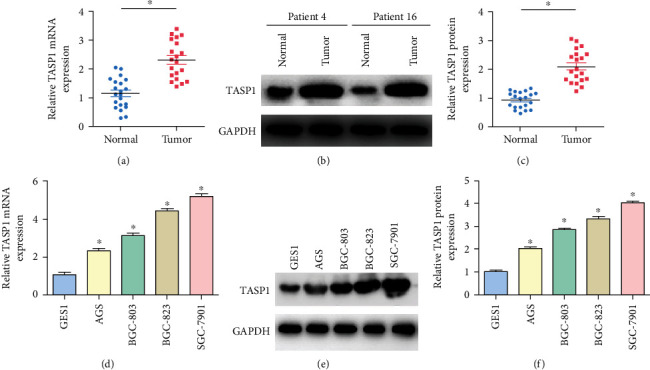
TASP1 is overexpressed in GC tissues and GC cells. (a) The expression of TASP1 mRNA in GC tissues and matched normal tissues via qRT-PCR (^∗^*p* < 0.05). (b) The expression of TASP1 protein in tissues from patient 4 and patient 16. (c) The expression of the TASP1 protein in GC tissues of 20 patients. (d) The TASP1 mRNA expression in GES1 cells and GC cells (BGC-803, AGS, SGC-7901, BGC-823) (^∗^*p* < 0.05). (e, f) The TASP1 protein expression in GES1 cells and GC cells (BGC-803, AGS, SGC-7901, BGC-823) (^∗^*p* < 0.05).

We firstly detected the expression of TASP1 mRNA and protein in the tissues of 20 GC patients and different GC cells. It was found that TASP1 mRNA levels in the tissues of GC patients were significantly higher when comparing with those in normal tissues (Figure 1(a)). Furthermore, the expression of the TASP1 protein in tumor tissues of 20 patients was higher than that in normal tissues (Figures 1(b) and 1(c)). The TASP1 mRNA levels were significantly higher in GC cells than those in control (Figure 1(d)). TASP1 protein levels were higher in GC cells than those in control, and the expressions of TASP1 in SGC-7901 cells and BGC-823 cells were higher than those in BGC-803 cells and AGS cells (Figures 1(e) and 1(f)). These data suggest that TASP1 is highly expressed in GC.

### 3.2. TASP1 Promotes the Proliferation of GC Cells

**Figure 2:**
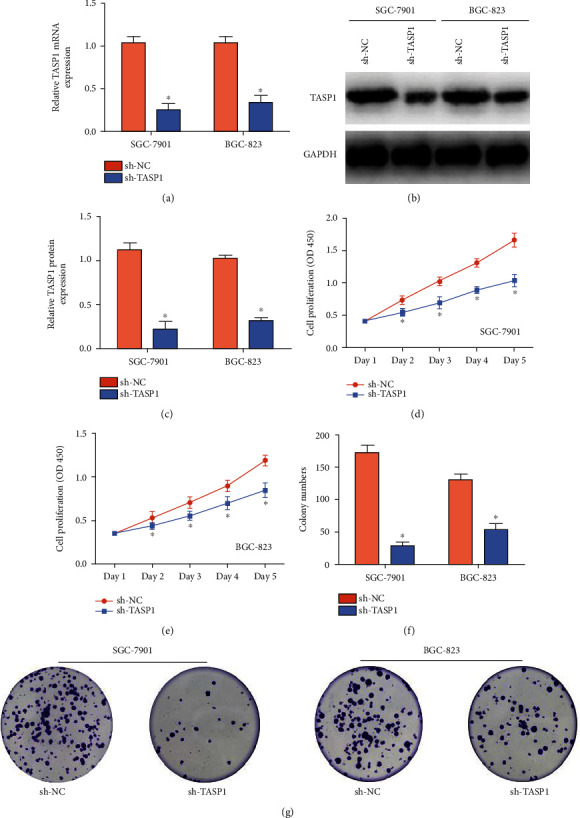
TASP1 promotes the proliferation of GC cells in vitro. (a) The TASP1 mRNA levels in SGC-7901 cells and BGC-823 cells transfected with sh-TASP1 (^∗^*p* < 0.05). (b, c) The levels of the TASP1 protein in SGC-7901 cells and BGC-823 cells transfected with sh-TASP1 (^∗^*p* < 0.05). (d, e) The proliferation ability of SGC-7901 cells and BGC-823 cells that were transfected with sh-TASP1 (^∗^*p* < 0.05). (f, g) Analysis of the number of GC colonies and their statistical significance (^∗^*p* < 0.05).

To explore the carcinogenic effect of TASP1 on GC, we chose SGC-7901 cells and BGC-823 cells with higher TASP1 expression for subsequent studies. As shown in Figures 2(a)–(c), TASP1 mRNA and protein levels reduced significantly in SGC-7901 cells and BGC-823 cells transfected with sh-TASP1. CCK-8 experiment was conducted to show that the proliferation abilities of SGC-7901 cells and BGC-823 cells transfected with sh-TASP1 were significantly reduced than those in SGC-7901 cells and BGC-823 cells transfected with sh-NC (Figures 2(d) and 2(e)). And TASP1 knockdown significantly reduced the colonies of SGC-7901 cells and BGC-823 cells (Figures 2(f) and 2(g)). Collectively, it is revealed that the TASP1 promotes GC proliferation in vitro.

### 3.3. TASP1 Promotes the Migration of GC Cells

**Figure 3:**
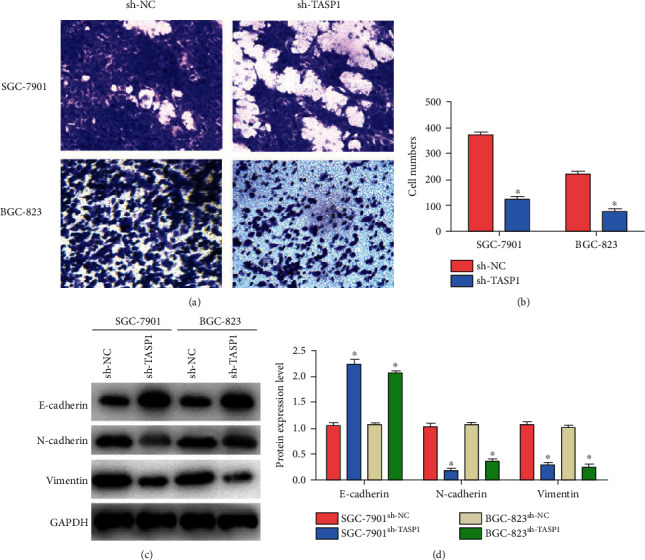
TASP1 promotes the migration of GC cells by regulating the levels of E-cadherin, N-cadherin, and vimentin. (a, b) Imaging analysis of the migration ability of SGC-7901 cells and BGC-823 cells transfected with sh-TASP1 and evaluating their statistical significance based on the numbers of migrated cells (^∗^*p* < 0.05). (c, d) Expression levels of E-cadherin, N-cadherin, and vimentin in SGC-7901 cells and BGC-823 cells transfected with sh-TASP1s (^∗^*p* < 0.05).

To explore how TASP1 controls the migration of GC cells, we conducted the transwell assay in vitro. Compared with SGC-7901 cells and BGC-823 cells transfected with sh-NC, SGC-7901 cells and BGC-823 cells transfected with sh-TASP1 significantly decreased the migration ability (Figures 3(a) and 3(b)). Large numbers of studies have reported that EMT mediates cancer invasion and metastasis [22]. We detected the expressions of E-cadherin, N-cadherin, and vimentin by western blot. It is found that the levels of E-cadherin of GC cells transfected with sh-TASP1 were higher, and the levels of N-cadherin and vimentin of GC cells transfected with sh-TASP1 were lower when compared with normal cells (Figures 3(c) and 3(d)). It suggests that TASP1 can promote GC migration in vitro.

### 3.4. TASP1 Promotes Development of GC Cell via the AKT/p-AKT Pathway

**Figure 4:**
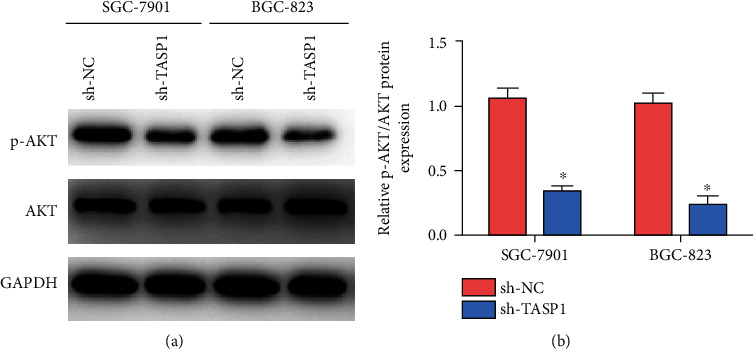
TASP1 promotes the phosphorylation of AKT in GC cells. (a) Western blot detected the expressions of AKT and p-AKT in GC cells transfected with sh-TASP1. (b) The relative p-AKT/AKT protein expression in GC cells transfected with sh-TASP1.

**Figure 5:**
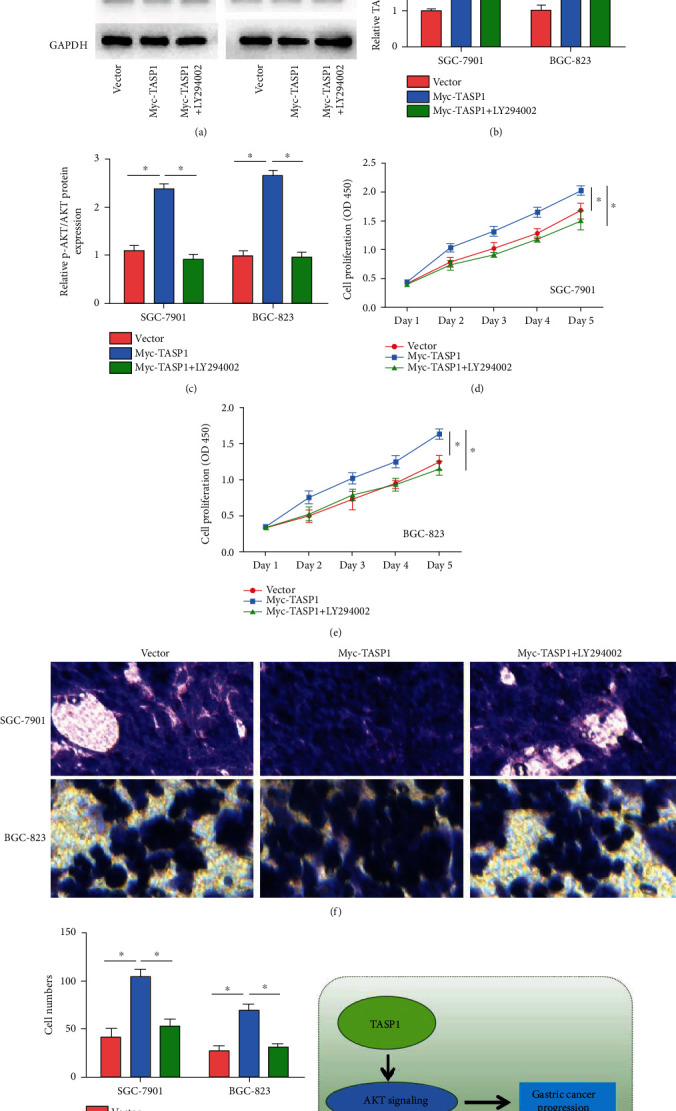
TASP1 promotes GC progression via the p-AKT/AKT signaling pathway. (a) The TASP1, AKT, and p-AKT protein expressions in Myc-TASP1-transfected GC cells that treated with LY294002. (b, c) The relative TASP1 and p-AKT/AKT protein expressions in Myc-TASP1-transfected GC cells that treated with LY294002. (d, e) The proliferation abilities of cells in Myc-TASP1-transfected GC cells that treated with LY294002. (f, g) The migration abilities of cells in Myc-TASP1-transfected GC cells that treated with LY294002. (h) The proposed mechanistic scheme of TASP1 regulating GC progression via AKT signaling.

To study the molecular mechanism of TASP1 in GC, we detected the p-AKT/AKT protein. TASP1 knockdown significantly decreased the expression of p-AKT in SGC-7901 cells and BGC-823 cells (Figures 4(a) and 4(b)). The TASP1 overexpression significantly increased the expressions of TASP1 and p-AKT in SGC-7901 cells and BGC-823 cells, and the supplement of AKT/p-AKT inhibitor LY294002 in TASP1-overexpressing GC cells did not change the TASP1 expressions but decreased the expressions of p-AKT (Figures 5(a) and 5(b)). The TASP1 overexpression promoted the proliferation and migration abilities of GC cells, but the proliferation and migration abilities of TASP1-overexpressing GC cells treated with LY294002 were significantly reduced than those in TASP1-overexpressing GC cells (Figures 5(c)–(f)). These data suggest that the p-AKT/AKT signaling may be involved in the process of GC mediated by TASP1 (Figure 5(f)).

## 4. Discussion

TASP1 is an endopeptidase with an asparaginase-2 homology domain, which recognizes and cleaves the substrate with a conserved peptide motif of aspartic acid at the P1 position [23]. TASP1 is not a traditional oncogene, but it can help the occurrence of cancer by cleaving MLL and TFIIA and be a potential anticancer drug target [13, 24]. In this work, it is the first time to report that the levels of TASP1 in GC patients tissues and GC cells are significantly upregulated. Similarly, previous studies showed that TASP1 was also increased in brain cancer, breast cancer, colon cancer, head and neck squamous cell carcinoma, and blood cancer [12].

Next, we observed that TASP1 could promote GC proliferation and migration. TASP1 knockdown inhibited the proliferation of GC cells. And TASP1 knockdown increased the E-cadherin expression and inhibited N-cadherin expression, thus inhibiting the migration of GC cells. Then, it was found that TASP1 knockdown reduced the expression of the p-AKT/AKT protein. These results indicate that TASP1 may promote GC proliferation and migration through the AKT/p-AKT pathway. In previous studies, Dong [16] found that TASP1 promotes the proliferation of breast cancer cells. Zhang [25] found that TASP1 promoted gallbladder cancer proliferation and migration. And it was found that p-AKT was in higher activated state in breast cancer, ALT cancer, liver cancer, lung cancer, and other malignant cancers, and high p-AKT was associated with the cancer development [26–29]. It suggests that TASP1 might be involved in the GC development.

This work indicates the role of the TASP1 expression in GC. However, there are some limitations here, such as small sample size, the relationship between TASP1 expression, and the stages in GC, and the therapeutic potential of TASP1 regulator in GC is unclear. In subsequent works, we will increase the numbers of sample. The gastric tumor slides were made to do the IHC staining of the TASP1. And we will explore the expression of TASP1 in different stages of GC. The therapeutic effect of TASP1 inhibitors on GC patients will also be studied.

In summary, TASP1 may be an effective marker for GC and a new target for GC treatment.

## Data Availability

All data are available upon request.
